# A Probable Association of Aseptic Meningoencephalitis, Complicated With Cerebral Salt Wasting Syndrome Following COVID-19 Vaccination: A Case Report

**DOI:** 10.7759/cureus.41106

**Published:** 2023-06-28

**Authors:** Ramanathan Ramesh, Arulmoly Kanagasingam, Sithy Sabrina, Uthayakumar Anushanth

**Affiliations:** 1 Medicine, Teaching Hospital Batticaloa, Batticaloa, LKA

**Keywords:** pfizer-biontech, hyponatremia, meningoencephalitis, cerebral salt wasting syndrome, covid 19

## Abstract

Coronavirus disease-19 (COVID-19) pandemic caused by the severe acute respiratory syndrome coronavirus 2 (SARS-CoV-2) occurred worldwide, and it affected millions of people around the world and killed millions of lives without a definitive treatment. During this challenging time, vaccine production has been hugely carried out leading to the invention of many vaccines against COVID-19. As any vaccine can have some side effects, COVID-19 vaccines also need surveillance and reporting side effects worldwide. Currently, more than 10 vaccines are available against SARS-CoV-2 infection globally. There are many neurological complications reported by SARS-CoV-2 vaccines. There are some reported neurological complications, such as ischemic stroke, Guillain-Barré syndrome, transverse myelitis, Bell’s palsy, cerebral venous sinus thrombosis, optic neuritis, meningoencephalitis, small fiber neuropathy, and Tolosa-Hunt syndrome. We present a case of an elderly man who presented with fever, fits, hyponatremia, and polyuria following COVID-19 vaccination and was found to have cerebral salt wasting (CSW) with the exclusion of other causes.

## Introduction

Hyponatremia is a common electrolyte imbalance presenting with fits, loss of consciousness, or poor response in elderly patients. It is important to find out the etiology of hyponatremia because the treatment varies depending on its cause. Early diagnosis and prompt treatment of hyponatremia reduce mortality and morbidity.

Cerebral salt wasting is an uncommon cause of hyponatremia compared to the syndrome of inappropriate antidiuretic hormone secretion (SIADH). Cerebral salt wasting is characterized by the renal loss of sodium leading to hypovolemia and hyponatremia. Cerebral salt wasting is most often caused by subarachnoid hemorrhage (SAH) [1], and there are other causes such as head trauma, infection (meningitis, encephalitis), tumors, and any brain insult that can result in cerebral salt wasting. Meningoencephalitis is a neurological condition resembling both meningitis, which is the inflammation of the meninges and encephalitis, which is the inflammation of the brain tissue. The pathophysiology of cerebral salt wasting is not well established, but there are two possible mechanisms contributing to cerebral salt wasting: the impaired sympathetic input to the kidney proximal tubules leading to the reduction of absorption of sodium and uric acid causes natriuresis and hypovolemia, and the second possible theory is that the circulating brain natriuretic peptide following insult to the brain decreases the reabsorption of sodium in the kidney [2,3]. COVID-19 vaccines are not previously recognized to cause cerebral salt wasting to explain the exact mechanism which caused cerebral salt wasting in our patient.

## Case presentation

He is a 69 years old, previously healthy man who presented with a history of fever for three days and two episodes of generalized tonic-clonic seizures which lasted for 5-10 minutes with urinary incontinence and post-ictal drowsiness, following a COVID-19 vaccination. He was admitted to the hospital fourth day of the standard dose of the second vaccination, and he received his first vaccine before seven months. He had a moderate fever, severe myalgia, and arthralgia for three days, and his symptoms were well responded to the paracetamol. There were no other seizure episodes after admission to the hospital. He did not have any history of urinary symptoms such as dysuria or hematuria and no history suggestive of respiratory tract infection, headache, or altered behaviors. He neither smokes nor takes alcohol. On admission to the hospital, he was febrile, drowsy, and dehydrated, and his capillary refilling time (CRFT) was prolonged. His cardiovascular system examination revealed tachycardia (110 bpm) with a low-volume pulse, and his blood pressure was 85/50. His respiratory and abdominal examinations were normal. On the first day of admission, he was persistently drowsy, and the next day, his consciousness improved almost to the normal level following initial resuscitation. His neurology showed nothing abnormal including an ophthalmoscopic examination. His brain imaging and non-contrast computer tomography failed to show evidence of any SAH or any other obvious intracranial pathology. His electrocardiogram showed sinus tachycardia without the evidence of any ischemia. His thyroid function test showed euthyroid status. His chest X-ray (CXR) did not reveal any abnormality, as his blood troponin I. His COVID-19 reverse transcription polymerase chain reaction (RT-PCR) also became negative. His investigation results are given in Table 1.

**Table 1:** Patient's investigation results are summarized. FBC: full blood count, WBC: white blood cells, Hb: hemoglobin, PLT: platelet, LFT: liver function test, AST: aspartate aminotransferase, ALT: alanine aminotransferase, ALP: alkaline phosphatase, ESR: erythrocyte sedimentation rate, CRP: C-reactive protein, RFT: renal function test, BU: blood urea, SE: serum electrolytes, UFR: urine full report, RBC: red blood cell, RBS: random blood sugar, CSF: cerebrospinal fluid, TB: tuberculosis, PCR: polymerase chain reaction.

Investigations	Day 1	Day 2	Day 3	On discharge
FBC-WBC/mm^3^	9.24×10^3^	8.2	9.3	7.2
Neutrophil (%)	68	72	78	65
Lymphocytes (%)	24	19	13	24
Eosinophils (%)	1.0	2.3	0.7	2.5
Hb (g/dl)	12.9	12.4	13.2	12.6
PLT mm3	196×10^3^	202	210	154
ESR	22		34	
CRP (mg/l)	24	18		16
LFT-AST (U/L)	34	54		52
ALT (U/L)	54	48		52
ALP (U/L)	104	115		98
Albumin (g/l)	29	30		32
Bilirubin-total (µmol/l)	10.2			
Direct bilirubin (µmol/l)	6.2			
Bilirubin- indirect (µmol/l)	4.0			
Serum osmolality (mosmol/l)	247		255	
Urine osmolality (mosmol/l)	121		104	
RFT-BU (mmol/l)	0.9		2.1	1.9
Serum creatinine (µmol/l)	66		66	68
SE-sodium (mmol/l)	114	121	135	138
Potassium (mmol/l)	3.9	3.2	3.3	3.8
Urine sodium (mmol/l)	186	178	100	64
UFR-pus cells	1-2	5-6		1-2
UFR-RBC	Nil	16-20		2-3
Protein	Nil	Nil		Nil
Organism	Nil	Nil		Nil
RBS (mmol/l)	4.6			
CSF full report				
Color	Clear			
Glucose (mmol)	4.0			
Protein (mg/dl)	38			
Cells-lymphocytes	5			
Polymorphs	Nil			
CSF gram stain	Negative			
CSF culture	No growth			
CSF TB-PCR	Negative			

While he was in the ward, he developed polyuria (150-200 ml/hour) despite his low blood pressure which required vasopressor support to maintain the blood pressure. Since his electroencephalogram (EEG) did not reveal any epileptic foci, we assumed that his fits and drowsiness were due to low sodium and started to evaluate the cause for hyponatremia while treating the patient. He was treated with initially hypertonic (3%) saline and followed by 0.9% saline. Also, we have started fludrocortisone 0.2 mg per day.

## Discussion

He was a 69-year-old man, apparently well before the history of COVID-19 vaccination. The following day of vaccination, he developed a fever, and it was assumed by the patient as a usual reaction to vaccination; he had taken paracetamol, and his fever and other symptoms settled. On the second day of fever, he developed two episodes of generalized tonic-clonic seizures with loss of consciousness and he was admitted to the emergency department. According to his presentation, we made a list of differential diagnoses with meningoencephalitis on top of the list. He had been started on intravenous ceftriaxone and intravenous acyclovir after getting blood and urine cultures and cerebrospinal fluid for full analysis. Meantime, we have started to investigate him to exclude other possible causes, such as head injury, sepsis, hypoglycemia, and electrolyte abnormalities. The non-contrast computed tomography (NCCT) of the brain, which was done on admission, did not show any obvious abnormalities. Because the magnetic resonance imaging (MRI) scan was not available in our institution, we did not perform it. All other possible causes were excluded as much as possible, such as hypoglycemia, and infections. All cultures were negative, and his chest X-Ray (CXR) was normal. As the patient's history and examination were not suggestive of infective endocarditis (IE), we did not perform the echocardiography. However, we could not proceed with the full viral polymerase chain reaction tests to exclude viral meningitis due to the poor resource setting in our region. On the serum electrolyte panel, we found severe hyponatremia of sodium level of 114 µmol/l with a normal potassium level. We initiated the investigations to find out any other etiology for his hyponatremia while managing the hyponatremia with 3% sodium chloride intravenous infusion carefully to prevent serious neurological complications. His cerebrospinal fluid (CSF) analysis failed to show evidence of central nervous system (CNS) infections. While in the ward, he did not develop any further fits, and with the correction of hyponatremia, his Glasgow Coma Scale (GCS) improved to 15/15. He was dependent on inotrope to maintain the mean arterial blood pressure above 65 mmHg. He developed polyuria while in the ward, and his average urine output was 150-200 ml/hour, despite his hypotension. His serum osmolality was 247 mmol/l, urine osmolality 380 mmol/l, and urinary sodium 180 meq/l with low serum sodium and uric acid concentration. All the above findings, particularly aseptic meningitis, and evidence of volume depletion with severe hyponatremia, and sequences of events following vaccination all guided us to arrive at the diagnosis of cerebral salt wasting rather than SIADH even without a full panel of viral studies [2,3]. As the management is entirely different between cerebral salt wasting (CSW) and SIADH, it is most important to differentiate them to give the correct management. As we made the diagnosis of aseptic meningoencephalitis complicated with cerebral salt wasting, the patient had been treated with initially hypertonic saline (3%) followed by normal saline (0.9%) and fludrocortisone (0.2 mg/day). Fludrocortisone helps to reduce the renal loss of sodium, and his neurological condition significantly improved, and he recovered completely with the above management [4].

Cerebral salt wasting syndrome is an underrecognized but a frequent cause of hyponatremia. It occurs mostly in neurosurgical patients, particularly in patients with subarachnoid hemorrhage, head trauma, or cranial surgeries. In our patient, we could not find any obvious common causes for cerebral salt wasting syndrome other than the recent COVID-19 vaccination. Because we did not perform an MRI and panel of viral serology, and considering the clinical presentation and subsequent management, we can only say the probable association following vaccination. Vaccines against COVID-19 infection, which is associated with a myriad of neurological adverse events such as headache, weakness, and transient sensory symptoms, and there are some serious adverse events also reported like Guillain-Barré syndrome, functional syndrome, cerebral venous thrombosis, Bell’s palsy, and transverse myelitis [5,6]. However, cerebral salt wasting associated with the COVID-19 vaccine is rarely reported.

## Conclusions

After the pandemic of COVID-19, several vaccines were introduced within a short period of time, and the side effect profile of these vaccines is not fully evaluated. Even though there are various neurological complications reported following COVID-19 vaccines, cerebral salt wasting is rarely reported. CSW is an important differential diagnosis when a patient presented with hypovolemic hyponatremia in the absence of direct brain insult. Our case report is an example for the clinician to think about aseptic meningoencephalitis, and CSW can be associated with COVID-19 vaccination.
